# Bone mineral density, mechanical properties, and trabecular orientation of cancellous bone within humeral heads affected by advanced shoulder arthropathy

**DOI:** 10.1002/jor.24633

**Published:** 2020-03-08

**Authors:** Vilijam Zdravkovic, Rolf Kaufmann, Antonia Neels, Alex Dommann, Jürgen Hofmann, Bernhard Jost

**Affiliations:** ^1^ Department of Orthopaedics and Traumatology Kantonsspital St. Gallen St. Gallen Switzerland; ^2^ Center for X‐ray Analytics, Empa, Swiss Federal Laboratories for Materials Science and Technology St. Gallen Switzerland

**Keywords:** biomechanics, micro‐CT, mineral bone density (BMD), proximal humeral fractures, skeleton analysis, stemless total shoulder arthroplasty

## Abstract

The mechanical properties of cancellous bone in the humeral head are increasingly interesting due to the increased popularity of stemless prosthetic fixation in the cancellous bone of the metaphysis. Age or pathology‐related systemic osteoporosis, inactivity, or pathology of the shoulder joint may influence the primary bonding of implants that rely on good cancellous bone quality. We assessed the bone mineral density (BMD) and anisotropy using micro‐computed tomography (micro‐CT) (0.04 mm voxel size) and correlated the results with indentation load/displacement response. Resected parts of humeral heads (from patients undergoing total shoulder replacement, n = 18) were used as probes. The region of interest was defined as 2 mm medial from the resection plane, presuming that it mirrored the bone quality lateral to the resection plane. The indentation tests were performed with a large probe (diameter 10 mm) in a single destructive loading procedure. The BMD and trabecular orientation were determined by micro‐CT. Our results showed a correlation between the BMD and the slope of the load/displacement curve. Furthermore, the trabeculae were predominantly oriented orthogonal to the joint surface. In conclusion, the predominant factor determining the bone quality and mechanical resistance to pressure appears to be the BMD, while trabecular orientation could not be related to load/displacement response. Statement of clinical significance: Bone quality predominately determines the mechanical properties of cancellous bone. This might be crucial when prosthetic implants need to be anchored in metaphyseal bone. Therefore, clinical decision‐making processes should also include local BMD measurements.

## 1 INTRODUCTION

Mechanical bone properties are important in orthopedic surgery and traumatology as many procedures rely on the mechanical connection between the bone and implanted devices. Historically, the main research focus about bone properties was concentrated on long tubular bones. By introducing new implants that rely on stability and bonding in cancellous bone, the mechanical properties of cancellous bone might influence biological response with consequences on overall treatment results.

Total shoulder prosthesis with stemless humeral components is a new development that relies on uncemented pressfit fixation in the cancellous bone of the humeral metaphysis, preserving the bone, and sparing the humeral canal. Furthermore, this avoids stem‐related complications such as intraoperative humeral fractures, stem loosening, stress‐shielding, and postoperative periprosthetic fractures^1^ as well as difficult stem extraction in cases of revision.

Computed tomography (CT) can be used to assess cancellous bone quality by measuring the local cancellous bone mineral density (BMD), for example, with peripheral quantitative CT (PQCT). Low BMD influences the failure rate after proximal humeral fracture fixation with locking plates, and the suggested threshold was found to be less than 95 mg/cm^3^.^2^ The cortical thickness ratio of the proximal humeral diaphysis in standard anteroposterior X‐rays is another valuable radiographic parameter to predict intraoperative bone quality in cases of proximal humeral fractures.^3^ Estimate of BMD is also used as an input parameter in the algorithm for treatment of proximal humeral fractures.^4^

Systemic measurement of BMD to predict mechanical bone response is not a practicable option for shoulder surgery. In cases of prosthetic replacement for arthritis or rotator cuff arthropathy, the local bone quality might be different from systemic BMD values due to inactivity. The surgeon has one final option to assess bone quality—intraoperative haptic assessment by pressing the cancellous bone surface, for example, with the thumb. However, Tulner et al^5^ showed that there was consensus among surgeons about haptic characteristics of cancellous bone in the humeral head at the resection plane.

Cancellous bone of the hip shows compression and distraction trajectories (trabecular pattern) but in the humeral head, there is no clearly visible anatomic response to loading forces. A theoretical model that accurately explains the mechanical properties of the proximal humerus could be useful to better understand the pathophysiology of trauma and mechanical complications after shoulder surgery. Finite element (FE) models have already been published and studied for other bones like the proximal tibia^6^, ^7^ and proximal humerus.^8^, ^9^, ^10^ However, all models rely on PQCT imaging which has insufficient resolution to determine the trabecular bone structure. Furthermore, mechanical properties are tested on anatomic specimens and not on bone samples taken from patients. In the proximal humerus, this could be an important factor because osteoarthritis or inactivity alone might change bone quality and/or structure (reduced BMD, the formation of bone cysts). Therefore, measurements and FE models calculated for normal bone (or bone of unknown status) might not be applicable to bone encountered in patients. Typically, the indentation measurements and FE models include subchondral cortical bone, but this part of the bone gets removed when implanting shoulder prostheses. Therefore, the probe does not usually reach the cancellous bone at the center of the humeral head.

The aim of our study was to determine how BMD affects mechanical properties of cancellous bone in the humeral head, especially in cases of known pathology potentially affecting the bone structure. We hypothesized that reduced BMD results in less resistance to penetrating the bone structure and that the trabecular orientation is mainly perpendicular to the joint surface.

## 2 METHODS

### 2.1 Specimens

The study was performed on 19 resected humeral heads from patients who had undergone a shoulder replacement. Inclusion criteria were: the patient had been scheduled for shoulder prosthesis and had signed informed consent. There were no age limitations. One sample had to be discarded from further analysis since the bone structure was altered due to aseptic bone necrosis. Eleven patients had osteoarthritis related to cuff tear arthropathy, five were classified as idiopathic primary osteoarthritis, and two as posttraumatic arthritis. The bone specimens were fixed in formalin. After nondestructive micro‐CT imaging, the specimens were destructively tested by indentation. The study was approved by the Institutional review board (IRB): Ethical Committee of Kanton St. Gallen, Switzerland (Ref. BASEC Nr. 2015‐00142).

### 2.2 Micro‐CT imaging, measuring BMD, and analyzing trabecular structure

**Figure 1:**
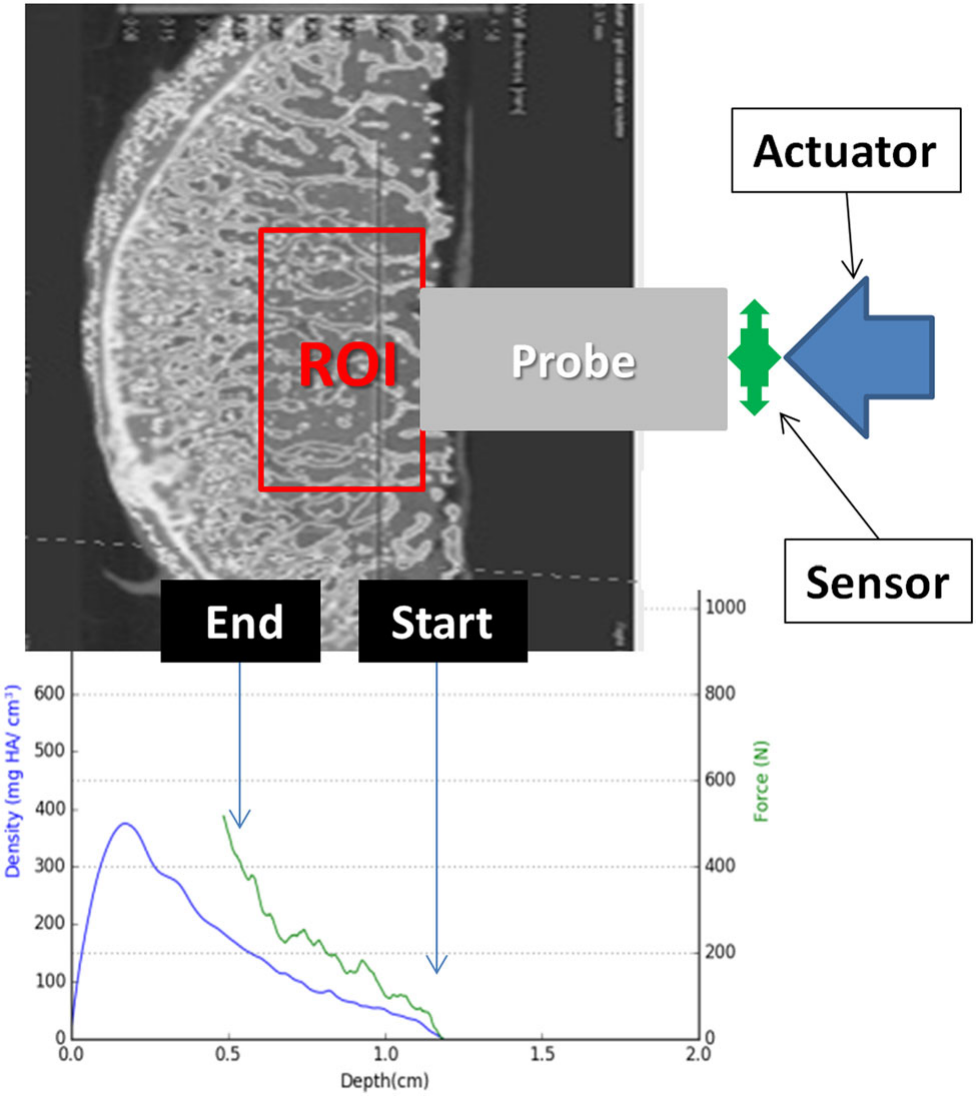
Schematic presentation of the experimental setup. Above: Placement of the region of interest (ROI) in the resected calotte of the humeral head. The probe is penetrating from left to right. Below: Combined graphical presentation of bone mineral density from micro‐CT (blue line) and load‐displacement curve (green line). The curves are running also from left to right according to the direction of penetration. CT, computed tomography [Color figure can be viewed at wileyonlinelibrary.com]

A micro‐CT with a voxel size of 40 µm was acquired from all samples. Comparing the gray values in the cancellous bone with gray values of a CT phantom of known densities (Type KP70, QRM GmbH, Moehrendorf, Germany), the mean bone density in the region of interest (ROI) in the cancellous bone could be calculated.^11^ The placement of the ROI presented in Figure 1.

The micro‐CT scans were performed at 140 kV and 70 µA. The setup consists of a Viscom Tube XT160, a Perkin Elmer flat panel detector (Model 1621) with 200 µm pixel size and a self‐built mechanical system for sample positioning and rotation.

After segmentation of the individual trabeculae, the data were also used to calculate mean orientation, thickness, length distribution, and amount of bifurcations in specific areas (skeleton analysis using VG Studio Max 3.0 Fiber Composite Material Analysis Module).^12^, ^13^

### 2.3 Indentation testing

**Figure 2:**
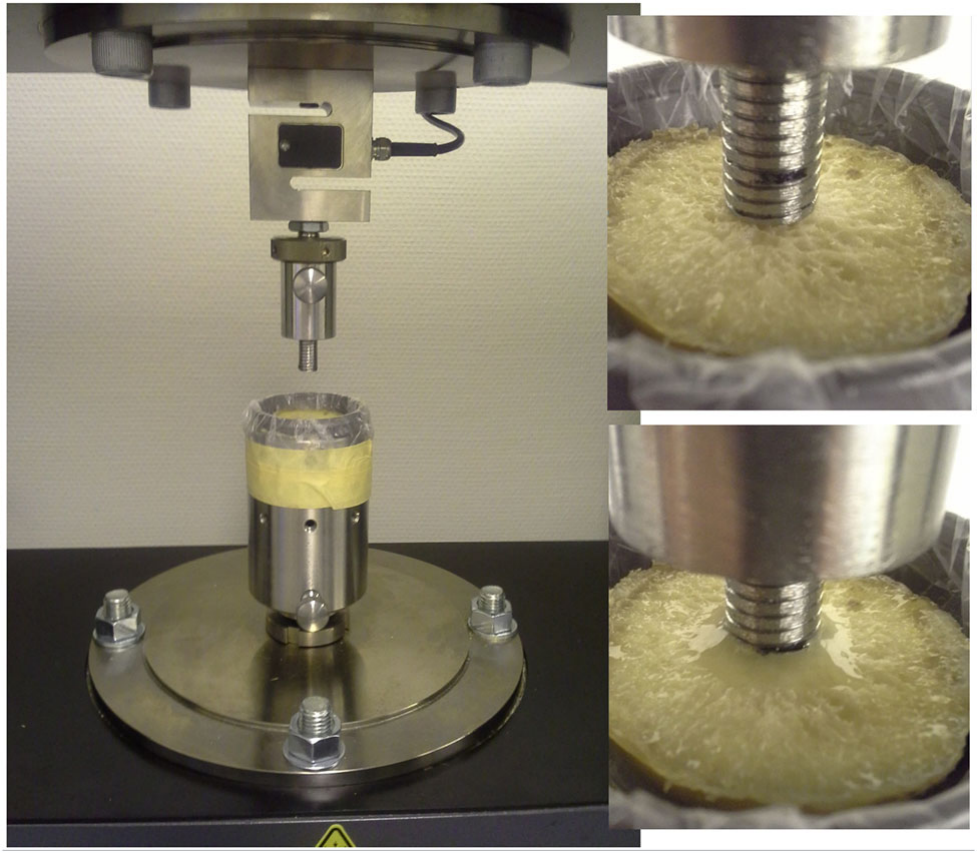
Experimental setup; custom‐made holder and custom‐made probe [Color figure can be viewed at wileyonlinelibrary.com]

Force‐displacement measurement was performed using material testing equipment (Zwick/Roell, Ulm, Germany). The probe was custom‐made (*d* = 10 mm) and fixed on the holder with a sensor (Figure 2).

**Figure 3:**
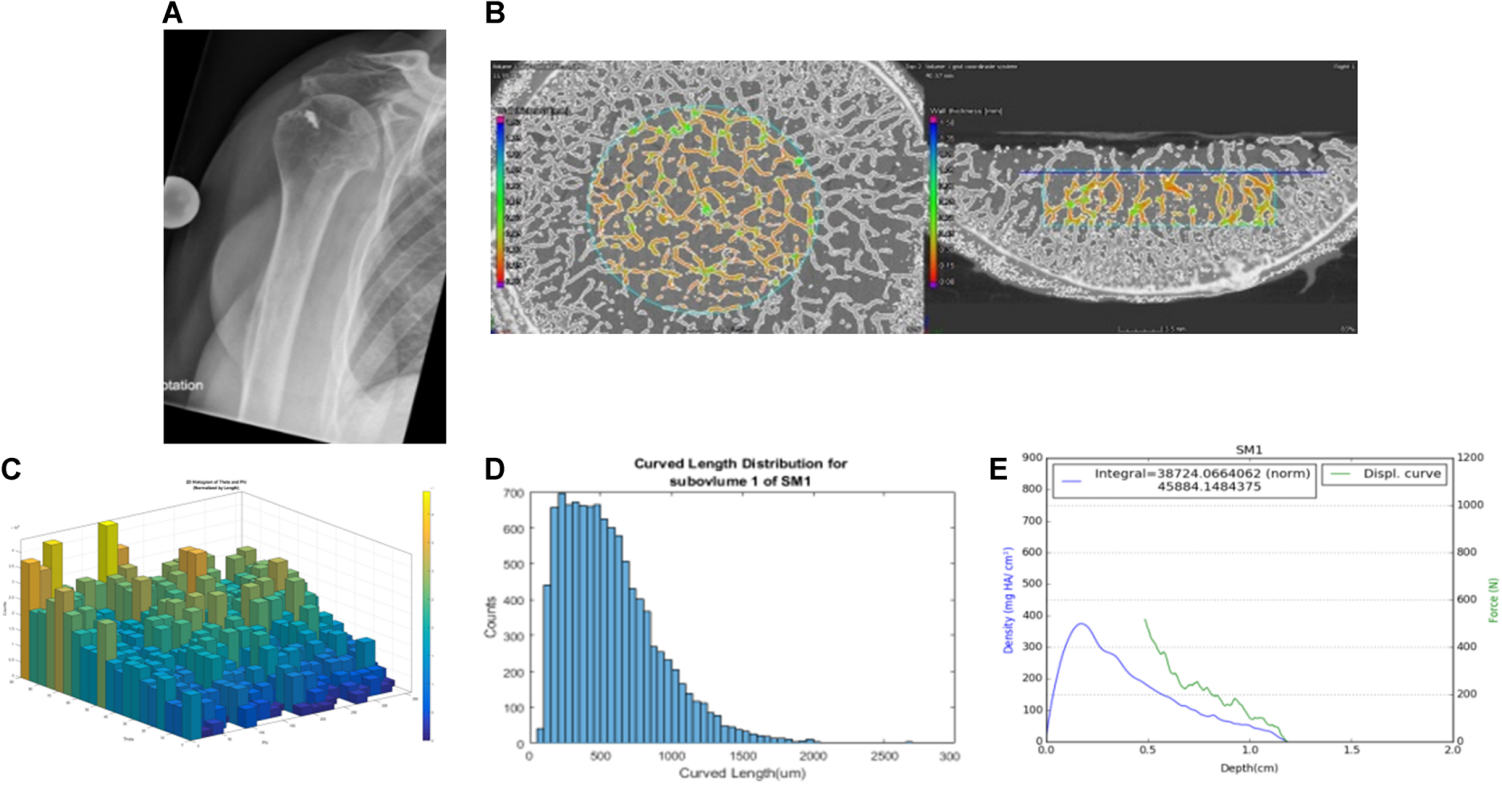
Presentation of data and images collected for each specimen. A, X‐ray; B, micro‐CT; C, trabecular orientation; D, length distribution; and E, load‐displacement curve combined with BMD curve. BMD, bone mineral density; CT, computed tomography [Color figure can be viewed at wileyonlinelibrary.com]

The specimens were fixed with 2K epoxy glue in a custom‐made holder. The indentation protocol was simple loading up to failure or stopped when a force of 1000 N was reached or when the indentation path extended over 7 mm. The resulting force‐displacement curve is shown together with the local density of the cancellous bone for sample number 15 in Figure 3. Such plots were created for all samples.

### 2.4 Numerical analysis

For numerical analysis of the relation between BMD and force‐displacement response, we constructed a factor representing the bone resistance to penetration of the probe. The force values until 0.5 mm probe penetration were discarded because the bone at the resection plane was potentially altered mechanically by the bone saw. A second‐order polynomial function was fitted over force/displacement values from 0.5 mm to 6 mm penetration (or at the depth achieved at maximal pre‐set force). The slope of the line (in N/mm) is taken as a constant (slope constant = SC) representing the response of the particular bone specimen to loading during indentation.

The values of BMD and SC for all specimens were entered in a table, represented graphically, different linear and exponential curves fitted and finally the curve with best *R* ^2^ value chosen for further interpretation.

## 3 RESULTS

**Figure 4:**
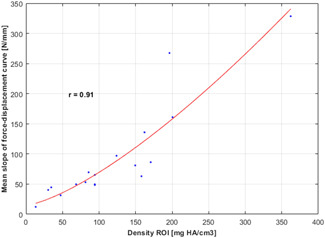
Curve of the function *SC* *=* a*BMD ^2^ + *c* fitted over BMD and SC values. BMD, bone mineral density; SC, slope constant [Color figure can be viewed at wileyonlinelibrary.com]

The results of BMD measurement and indentation measurements for all specimen are presented in Table 1. The average BMD was 120.4 mg HA/cm^**3**^ (range, 13.3‐362.2). Average SC was 93.6 N/mm, (range, 12.2‐328.5 N/mm). The best fitting of BMD/SC values was found for the square function (Figure 4):

$$\mathrm{SC}=a*\mathrm{BM}D^{2}+c$$

**Table 1:** Results of BMD measurement and slope constant (SC) of force‐distance curve obtained by indentation measurements

Specimen	Diagnosis	Slope constant	Density ROI
		N/mm	(mg HA/cm^3^)
SM1	CTA	44.6	34.6
SC3	CTA	49.7	68.6
BA6	CTA	48.6	94.2
BM9	CTA	69.5	85.7
PJ14	CTA	63	158
15	CTA	65.2	94.2
16	CTA	40.3	30.4
17	CTA	86.3	170.7
20	CTA	12.2	13.3
KV10	CTA	49.6	94.2
PH11	CTA	81	149.5
RW4	OMA	161	200.5
BM2	OMA	97	123.9
SE7	OMA	53.1	81.4
IJ8	OMA	31.4	47.4
18	OMA	267.5	196.3
AA13	PTOMA	328.5	362.2
DR12	PTOMA	135.9	162.2

Abbreviations: BMD, bone mineral density; CTA, arthritis due to cuff tear arthropathy; OMA, primary idiopathic arthritis; PTOMA, posttraumatic arthritis; ROI, region of interest.

The correlation coefficient was 0.91.

It could be shown that in most humeral heads there was a small tendency for trabecula orientation orthogonal to the humeral head surface, but it was not very pronounced (Figure 3). All the other parameters (bifurcations, length, and thickness of the trabeculae) revealed no tendencies in their distributions across the ROI.

## 4 DISCUSSION

Regional or local BMD, in general, is an important factor to be considered in orthopedic surgery while determining treatment strategy. Also, in the course of operation the information about BMD may be useful when the mechanical stability of implanted devices is questioned. In our study, we investigated BMD in the proximal humerus in the light of applying and potentially developing stemless prosthetic designs. Improved knowledge about cancellous bone may also allow a better understanding of fracture patterns in the proximal humerus, providing useful information for further osteosynthesis implant designs.

Our results indicate that BMD is the predominant factor for mechanical stability in terms of resisting penetration. Although not anisotropic, the direction of trabeculae did not correlate to load/displacement response. There are no comparable results in the literature because recent similarly designed studies^6^, ^10^ also involved subchondral bone (Table 2). Our focus was clearly on the cancellous bone because some of the stemless prosthetic designs only contact the resection plane without reaching the cortical ring. It could be perceived that the best and most uniform force transition between the relatively stiff element (anchor and the base plate of the prosthesis) and plastic/elastic element (cancellous bone) can be achieved if the second element has uniform density. Any peaks of density (eg, calcification in the structure or cortical rim at the periphery) may induce torsional moments that may lead to the micromotion or rocking of the implant and consequently primary nonbonding. A precise and validated FE model would allow a better understanding of bone/implant interaction, which is one of our future study intentions.

**Table 2:** Comparison of studies by Nazemi et al^6^ and Zumstein et al^10^ with the present study

	Nazemi et al	Zumstein et al	Present study
Structure	Subchondral	Subchondral	Cancellous
Bone	Proximal tibia	Humerus	Humerus
N spec/n patients	11/8	32/?	18/18
Conservation	Fresh frozen	Formaldehyde	Fresh in formaldehyde
Age/sex information	Yes	No	Yes
Mean age	76	80	71
Pathology	No	No	Yes
QCT slice thickness	0.5	0.6	0.04
Probe diameter (mm)	3.5	1.3	10
Max force applied (N)	250	?	1000

Abbreviation: QCT, quantitative computed tomography.

BMD appeared to vary relatively widely in our study, which may partially be explained by variation in pathologies (primary osteoarthritis, secondary [posttraumatic] osteoarthritis, and cuff tear arthropathy). These different pathologies have intentionally been included in order to assess the potential variability of BMD allowing for larger application of the study results. However, this compromises the power of potential diagnosis‐related subgroup analyses which was therefore not performed. We observed that in some specimens bone cysts or apparently amorphous and dense bone areas influenced the outcome of BMD. This makes the outcome sensitive to the placement of ROI, but also illustrates the real live application circumstances that could not be assessed using anatomical specimens. Our ROI is large enough to permit calculations that contain complete cysts or dense areas.

The anatomical stemless prosthetic design has been introduced without prior extensive research of the microstructure of cancellous bone, as in the present study. Although short to midterm results appear promising,^14^ long term results have yet to be observed. Further developments like inverse stemless shoulder prostheses might induce even higher stress on implant/bone interface and rely much more on primary fixation stability. Therefore, the results of the present study might help avoid the critical design drawbacks of new implants. Furthermore, our results could be projected onto other bones and pathologies where the interest is focused on the cancellous bone (eg, spine, especially related to osteoporotic fractures).

Our study has some limitations. The ROI is not exactly where stemless prostheses are actually placed. It was placed on “the other side” of the bone surface, and then further 0.5 mm deeper below the cut plane. This security margin of 0.5 mm was necessary to avoid a layer of cancellous bone whose structure is potentially damaged by the saw. Another limitation is the destructive nature of indentation tests using one large probe, which limits the testing to only one assay. We could have tested more spots on the specimen surface, map the results. and calculate average values using a small probe. We accepted this disadvantage in favor of applying a construction similar to the base plate of a stemless prosthesis. The next limitation is also related to prosthetic design: we did not measure pull‐out strength or resistance to horizontal torsional loading, which might be of interest for prosthetic anchors, cages, or screws. However, an FE model could provide these answers in the future, with detailed micro‐CT based structural information and the results of indentation tests. At present, the stemless design is mainly available for anatomic shoulder arthroplasty *but there is a trend to implement stemless design for reverse shoulder arthroplasty as well. Therefore, we included also patients with cuff tear arthropathy, but this introduced further limitations to our study: small sample size and heterogeneities of pathologies*.

In conclusion, the predominant factor determining the bone quality and mechanical resistance to pressure appears to be the BMDs, while the trabecular orientation could not be related to load/displacement response.

## CONFLICT OF INTERESTS

The authors declare that there are no conflict of interests.

## AUTHOR CONTRIBUTIONS

The authors VZ, RK, and JH substantial contributed to research design, the acquisition, analysis, and interpretation of data; AN analyzed the data and revised the manuscript critically; BJ and AD substantially contributed to interpretation of the data and revised and approved the final version. All authors have approved the final version of the manuscript.

## References

[1] Churchill RS . Stemless shoulder arthroplasty: current status. J Shoulder Elb Surg. 2014;23:1409‐1414. 10.1016/j.jse.2014.05.005 25027481

[2] Krappinger D , Bizzotto N , Riedmann S , Kammerlander C , Hengg C , Kralinger FS . Predicting failure after surgical fixation of proximal humerus fractures. Injury. 2011;42:1283‐1288. 21310406 10.1016/j.injury.2011.01.017

[3] Spross C , Kaestle N , Benninger E , et al. Deltoid tuberosity index: a simple radiographic tool to assess local bone quality in proximal humerus fractures. Clin Orthop Relat Res. 2015;473:3038‐3045. 25910780 10.1007/s11999-015-4322-x PMC4523505

[4] Spross C , Meester J , Mazzucchelli RA , Puskás GJ , Zdravkovic V , Jost B . Evidence‐based algorithm to treat patients with proximal humerus fractures‐a prospective study with early clinical and overall performance results. J shoulder Elb Surg. 2019;28:1022‐1032. 10.1016/j.jse.2019.02.015 31003888

[5] Tulner SAF , Zdravkovic V , Külling F , Jost B , Puskas GJ . Haptic assessment of bone quality in orthopedic surgery: no consensus but perspective for high training potential. Int J Med Educ. 2017;8:437‐438. 29278525 10.5116/ijme.5a38.168c PMC5768440

[6] Nazemi SM , Amini M , Kontulainen SA , et al. Optimizing finite element predictions of local subchondral bone structural stiffness using neural network‐derived density‐modulus relationships for proximal tibial subchondral cortical and trabecular bone. Clin Biomech. 2017;41:1‐8. 10.1016/j.clinbiomech.2016.10.012 27842233

[7] Nazemi SM , Kalajahi SMH , Cooper DML , et al. Accounting for spatial variation of trabecular anisotropy with subject‐specific finite element modeling moderately improves predictions of local subchondral bone stiffness at the proximal tibia. J Biomech. 2017;59:101‐108. 28601243 10.1016/j.jbiomech.2017.05.018

[8] van der Helm FC . A finite element musculoskeletal model of the shoulder mechanism. J Biomech. 1994;27:551‐569. 8027090 10.1016/0021-9290(94)90065-5

[9] Kennedy J , Feerick E , McGarry P , FitzPatrick D , Mullett H . Effect of calcium triphosphate cement on proximal humeral fracture osteosynthesis: a finite element analysis. J Orthop Surg. 2013;21:167‐172. 10.1177/230949901302100210 24014777

[10] Zumstein V , Kraljević M , Wirz D , Hügli R , Müller‐Gerbl M . Correlation between mineralization and mechanical strength of the subchondral bone plate of the humeral head. J Shoulder Elb Surg. 2012;21:887‐893. 10.1016/j.jse.2011.05.018 21872492

[11] Burghardt AJ , Kazakia GJ , Laib A , Majumdar S . Quantitative assessment of bone tissue mineralization with polychromatic micro‐computed tomography. Calcif Tissue Int. 2008;83:129‐138. 18685797 10.1007/s00223-008-9158-x PMC2801565

[12] Pothuaud L , Porion P , Lespessailles E , Benhamou CL , Levitz P . A new method for three‐dimensional skeleton graph analysis of porous media: application to trabecular bone microarchitecture. J Microsc. 2000;199:149‐161. 10947908 10.1046/j.1365-2818.2000.00725.x

[13] Doube M , Kłosowski MM , Arganda‐Carreras I , et al. Free and extensible bone image analysis in Image. J Bone. 2010;47:1076‐1079. 10.1016/j.bone.2010.08.023 PMC3193171 20817052

[14] Krukenberg A , McBirnie J , Bartsch S , et al. Sidus stem‐free shoulder system for primary osteoarthritis: short term results of a multicenter study. J Shoulder Elbow Surg. 2018;27:1483‐1490. 29625813 10.1016/j.jse.2018.02.057

